# Does the Shoe Fit? Real versus Imagined Ecological Footprints

**DOI:** 10.1371/journal.pbio.1001700

**Published:** 2013-11-05

**Authors:** Linus Blomqvist, Barry W. Brook, Erle C. Ellis, Peter M. Kareiva, Ted Nordhaus, Michael Shellenberger

**Affiliations:** 1 Breakthrough Institute, Oakland, California, United States of America; 2 The Environment Institute and School of Earth and Environmental Sciences, The University of Adelaide, Adelaide, South Australia, Australia; 3 Department of Geography and Environmental Systems, University of Maryland Baltimore County, Baltimore, Maryland, United States of America; 4 The Nature Conservancy, Seattle, Washington, United States of America; University College London, United Kingdom

## Abstract

The global overshoot indicated by Ecological Footprint calculations consists entirely of an unreliable reframing of human carbon emissions and none of the five other land-use categories—cropland, grazing land, built-up land, fishing grounds, and forests. The Ecological Footprint is therefore “so misleading as to preclude its use in any serious science or policy context,” argue Blomqvist et al. in this perspective.

Despite its relative youth (less than two decades), the ecological footprint (EF) is a commonly used term in environmental science, policy discussions, and popular discourse. The motivation behind the concept is sound—we must account for, and quantify, the impacts of humanity on Earth's ecosystems if we are to manage the planet sustainably for the benefit of both human well-being and our natural heritage. The EF seeks to measure humanity's use of renewable biological resources, which can then be compared to the planet's capacity to regenerate these resources. The result of EF calculations that is quoted most widely is that humanity currently uses the equivalent of 1.5 Earths to support human needs. Therefore, we are already exceeding the planet's carrying capacity in what amounts to “ecological overshoot” [1],[2].

First popularized in the mid-1990s by Wackernagel and Rees [3], the EF has influenced the policies and communications of many governmental and non-governmental organizations. For example, EF metrics feature in the World Wildlife Fund's *Living Planet Report*, Worldwatch Institute's *State of the World*, the *Global Environment Outlook* of the United Nations Environment Program, the United Nations Development Program's *Human Development Report*, and the International Union for Conservation of Nature (IUCN)'s *Transition to Sustainability*; the Convention on Biological Diversity chose the EF as a key biodiversity indicator [4]–[9]. Given the broad influence and popular appeal of the EF, its measurement and underpinning assumptions warrant close scrutiny. Technical critiques of footprint methodology have been published [10]–[15], but footprint statistics continue to infuse policy discussions. Any global metric that attempts to capture and summarize a range of large-scale and complex phenomena is sure to entail simplifications, biases, errors, and gaps. Such limitations are unavoidable and must be traded off against the benefits, such as their utility for prioritization, target setting, and communication. This Perspective intends to demonstrate, however, that EF measurements, as currently constructed and presented, are so misleading as to preclude their use in any serious science or policy context. Drawing from these findings, we outline a set of principles that any ecological indicator ought to consider in order to be scientifically sound and relevant for policy.

## Measuring Footprint Size

The most widely accepted and published (in popular as well as peer-reviewed literature) EF comes from the Global Footprint Network, which has developed and published a standardized methodology [16]–[18]. This EF is what we examine in this article. Its methodology involves constructing and comparing two separate “accounts,” representing the supply and demand of renewable biological resources across six mutually exclusive land-use types: cropland, grazing land, forest, fishing ground, built-up land, and the area of forest required to offset human carbon emissions (the carbon footprint). The first account, the *ecological footprint of consumption*, is an estimate of the renewable biological resources required for consumption by a specified human population and for assimilation of its carbon wastes. The amount of biological productivity available within the six land-use types is termed *biocapacity*. Biocapacity and footprint of consumption are both converted into an abstract land unit (global hectares or gha), representing the bioproductivity of a world-averaged hectare [15],[16]. On the global scale, when the footprint of consumption exceeds biocapacity, the interpretation is that humans are exceeding the regenerative capacity of Earth's ecosystems and therefore depleting stocks of natural capital, a state known as “overshoot” [19].

It is possible to apply the EF on a variety of spatial scales from cities and countries up to the global level. On a national scale, it compares the domestic footprint of consumption with domestic biocapacity. However, rather than indicating sustainability in the use of domestic or global biological resources, it is a measure of self-sufficiency [1],[11]. Hence, an ecological “deficit”—where domestic demand exceeds domestic supply—reflects patterns of trade that in themselves can be both positive and negative from an environmental viewpoint [20]. Therefore, in this Perspective, we focus only on the global level and on the assertion that humanity, as a whole, is in a state of planetary ecological overshoot.

## The EF and the Carbon Footprint

**Figure 1:**
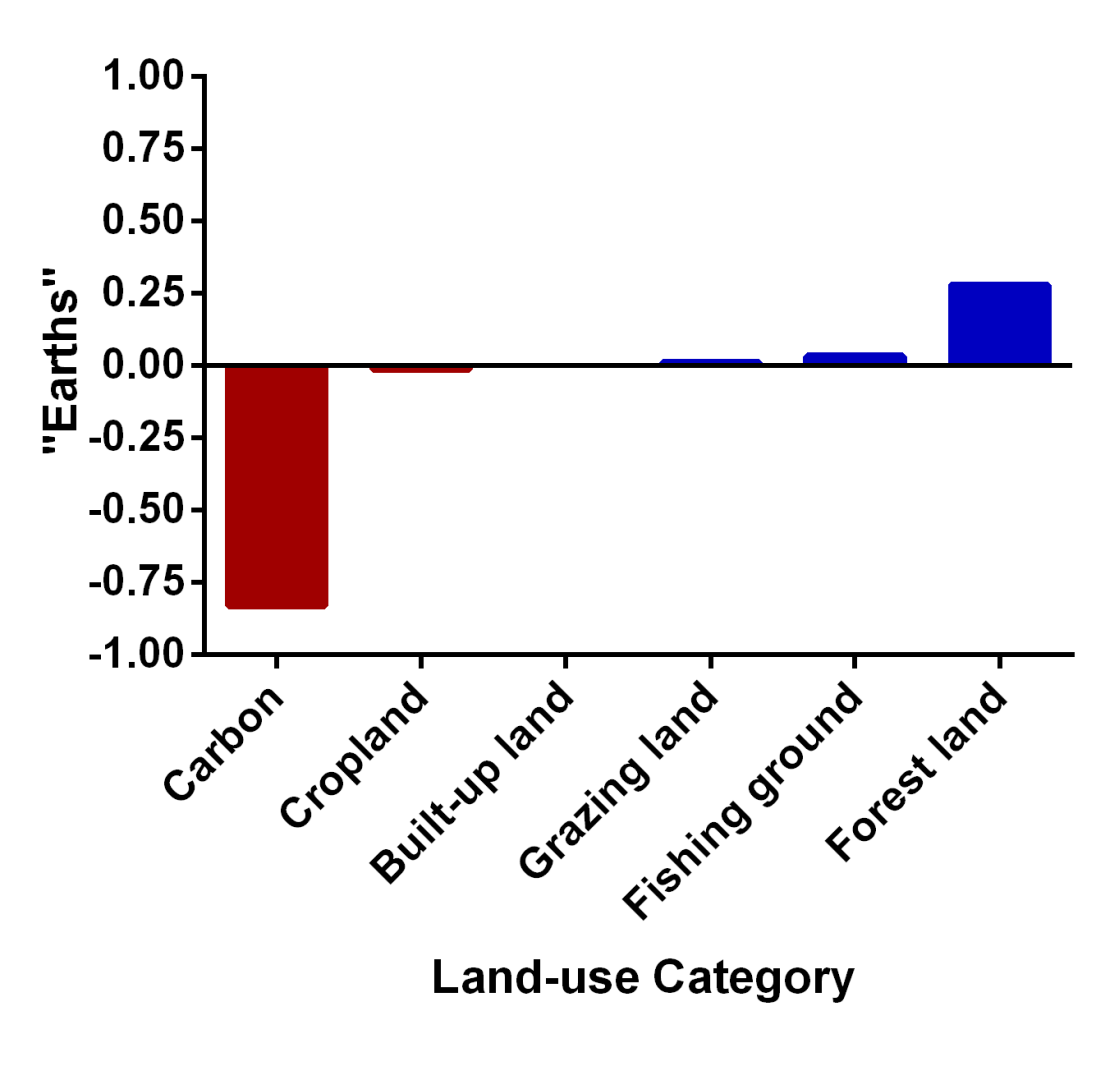
Net biocapacity (biocapacity minus footprint of consumption) by land-use category, shown as a fraction of total global biocapacity (one “Earth”) in 2008. Red bars indicate deficit, blue bars surplus. The sum of the net biocapacity of all land-use types is approximately −0.5, corresponding to the claim that humanity is using “1.5 Earths” worth of biocapacity every year [21].

The calculation of the footprint of consumption and biocapacity follows a distinct methodology for each land-use type that, along with an overview of the limitations of this type of analysis in assessing the sustainability of natural resource consumption, is outlined in Table 1. When the global EF is decomposed into its six components (Figure 1), none of the five non-carbon land-use categories has any substantial ecological deficit—suggesting that depletion of cropland, grazing land, forest land, fishing grounds, and built-up land is not occurring on an aggregate, global level. This result stems from the fact that the accounts for cropland, grazing land, and built-up land are constructed in such a way that they are always near equilibrium, with the footprint of consumption by definition nearly equal to biocapacity; fishing grounds and forest land are both in surplus (see explanations in Table 1). Hence, virtually all of the ecological overshoot comes from the EF's measure of the rate at which carbon dioxide is accumulating in the atmosphere. Indeed, if one excludes carbon, global biocapacity exceeds the footprint of consumption by about 45% in 2008 (the latest year for which data are available) and by an average of 69% over the period from 1961 to 2008. These figures appear to indicate a sustainable pattern of consumption, with productivity rising to meet growing demand [18],[21]. Another interpretation is that, beyond fossil-carbon waste, the EF is a poor representation of how well we are managing the planet, because a wide range of studies indicate that harm to Earth's ecosystem services is already significant, including declining soil fertility, increasing water scarcity, lowering of groundwater tables, oft-depleted fisheries, and loss of evolutionary history through species and population extinctions [22]–[26].

**Table 1:** Calculation of biocapacity and footprint of consumption in non-carbon land-use types.

Land-Use Category	Biocapacity	Footprint of Consumption	Comment
**Cropland**	Combined annual productivity (net growth) of all cropland.	Annual harvests (production) of primary and derived crop products.	Since biocapacity and footprint of consumption are by definition always roughly equal [18], the methodology cannot detect any substantial depletion or surplus of natural capital in croplands. Hence, the EF is currently unable to indicate the sustainability or unsustainability of this land-use category.
**Grazing land**	The amount of above-ground net primary production in grasslands per year.	Total annual feed requirement for livestock minus cropped feeds.	As with croplands, the footprint of consumption usually closely matches—and never exceeds—the biocapacity. The EF is, therefore, currently unable to indicate the sustainability or unsustainability of this land-use category.
**Forest**	Net annual increment of merchantable timber.	Annual harvests of fuelwood and timber to supply forest products.	The EF is able to register depletion or surplus of natural capital, in the form of wood biomass. Biocapacity has exceeded footprint of consumption by an average of 224% between 1961 and 2008 [21]. In other words, less than one third of annual growth in biomass is harvested for human use. Note, however, that the EF does not register declines in global forest area [47] or ongoing losses of primary forests in exceptionally biodiverse tropical regions [33].
**Fishing ground**	Total sustainably harvestable primary production per year, based on estimates of sustainable annual production converted to primary production by accounting for the trophic level of each harvested species, transfer efficiency of biomass between trophic levels, and the discard rate for bycatch.	Annual primary production required to sustain the harvested fish, converted to primary production in the same way as for biocapacity.	The surplus shown by the EF's thermodynamic methodology stands in contrast to other data on fisheries, with the FAO reporting 87% of stocks either fully exploited or overexploited [25]. As Kitzes et al. (2009) note, this category “ignore[s] the importance of availability and quality of fishing stocks (including large variation in harvest rates across different target species) in determining actual regenerative capacity in a given year.”
**Built-up land**	The area covered by human infrastructure, including transportation, housing, industrial structures, and reservoirs for hydroelectric power generation. Both the footprint of consumption and biocapacity of built-up land are defined as the bioproductivity of an equivalent area of cropland. This land-use category is always in equilibrium, since both quantities capture the amount of bioproductivity lost to encroachment by physical infrastructure [18].		The constant equilibrium of this component means that the EF is unable to illustrate the sustainability of this land-use type; neither about cities and infrastructure as such (they always count for the same), nor about the expansion of built-up land (one land-use type in equilibrium replaces another with no effect on the global ecological surplus or deficit).

## Determining the Size of the Carbon Footprint

Given that, as calculated by existing methods, humanity's global EF is practically equivalent to its carbon footprint, it is essential to determine just how humanity's carbon shoe size is measured. As assessed by the Global Footprint Network, the carbon footprint is the additional area of forest (expressed in gha) needed to sequester all net anthropogenic emissions of carbon dioxide (CO_2_) after subtracting the fraction of these estimated to be absorbed by oceans (currently 28%) [21]. (Long-lived greenhouse gases other than carbon, as well as greenhouse-gas emissions arising from land-use change, are not included in the analysis [16].) In other words, the EF defines carbon uptake in forests as the single mechanism for offsetting human emissions of greenhouse gases from industrial activity to the atmosphere. The exact formula for the carbon footprint is:
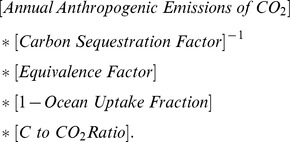



The terms after “Annual Anthropogenic Emissions of CO_2_” are the “footprint intensity of carbon” and equal roughly 0.25. This is the number of gha it takes to offset one ton of CO_2_. The number of additional real-world hectares of forest needed to offset the *entire* carbon footprint—if the definition of sustainability is zero net additions of CO_2_ to the atmosphere—is roughly 8 billion, corresponding to a little over half of the total land area of the Earth. From the formula, we can see that the carbon footprint area is essentially calculated by dividing total anthropogenic carbon emissions remaining after accounting for ocean uptake (i.e., 72% of net human emissions) by the rate at which existing forests sequester carbon. Therefore, the carbon footprint is inversely proportional to the assumed carbon sequestration rate in forests; double the biomass uptake rate and the carbon footprint is cut by half; halve it and the carbon footprint doubles. This single assumption is consequently what drives the conclusion that we have overshot the planet's capacity, and, as such, it should be scrutinized carefully.

The assumed carbon sequestration rate is reportedly based on a weighted average of the annual increment of merchantable timber per hectare in a sample of existing forest biomes. In the latest National Footprint Accounts, the rate is set to 0.97 metric tons of carbon (t C) per hectare per year (ha^−1^ year^−1^) [18],[21]. How robust is this estimation of global carbon uptake rates in existing forests? Given the extrapolations required to move from a hectare to a planetary scale, even minor variations to the assumed carbon sequestration rate would have a significant impact on the total size of the global EF. The large natural variability in carbon sequestration rates over time and space—and major uncertainties in their measurement—makes this extreme sensitivity a reason for caution [27]–[30]. The net uptake of carbon in terrestrial ecosystems has, over the past 5 decades, fluctuated between zero in some years to nearly 6 Gt C yr^−1^ in others [29]. This uncertainty is increasingly exacerbated by the effects of climate change, nitrogen deposition, and other forms of global change [31],[32]. If the world's forests were to become a net source of carbon later in this century—as some scenarios suggest [32]—the global EF would be infinite, since no amount of additional forest could suck the new additions of fossil carbon out of the air.

The additional amount of forest with world-average carbon uptake rate that would be required to completely offset human emissions of carbon to the atmosphere is therefore highly uncertain. More fundamentally, the very choice of offset mechanism to illustrate the carbon footprint is arbitrary. What exists in reality is a certain amount of emitted carbon that is absorbed neither by forests nor oceans and that therefore contributes to rising concentrations of carbon in the atmosphere. This amount has, in the past decade, fluctuated around 4 Gt C per year [29]. To illustrate the magnitude of this addition, the EF calculates the hypothetical area of forest with current world-average carbon sequestration rates that would be needed to fully offset this addition. But one might use, with equal validity, the area of *new* forest needed to offset these emissions. The only difference is in the figure for carbon sequestration plugged into the carbon footprint equation shown above. As a thought experiment, when plugging a carbon sequestration rate of 2.6 t C ha^−1^ year^−1^ or higher into the EF calculation, *the entire global ecological overshoot disappears*. As shown in Table 2, 2.6 t C ha^−1^ year^−1^ is a plausible expectation: many plantations, with different tree species and in different places, exceed this rate. Conversely, if the offset mechanism of choice were old-growth forests—an important target of conservation efforts [33], but for which respiration rates often equal sequestration—the net balance of carbon sequestration from forests is zero or close to it. This low biocapacity would drastically enlarge the total EF, which approaches infinity as the assumed carbon sequestration rate declines toward zero.

**Table 2:** Net carbon sequestration in forest plantations.

Climate Domain	Ecological Zone	Above-Ground Net Carbon Sequestration (t C ha^−1^ yr^−1^)
**Tropical**	Rain forest	7.1
	Moist deciduous forest	4.7
	Dry forest	3.8
	Shrubland	2.4
	Mountain systems	2.4
**Subtropical**	Humid forest	4.7
	Dry forest	3.8
	Steppe	2.4
	Mountain systems	2.4
**Temperate**	Oceanic forest	2.1
	Continental forest	1.9
	Mountain systems	1.4
**Boreal**	Coniferous forest	0.5
	Tundra woodland	0.2
	Mountain systems	0.5

*Note:* Approximate above-ground net carbon sequestration in forest plantations (t C ha^−1^ yr^−1^) by ecological zone, as reported in the 2006 IPCC Guidelines for National Greenhouse Gas Inventories [48].

These examples demonstrate that only slight adjustments to the assumed carbon sequestration rate can produce wildly different outcomes, ranging from global ecological surplus to infinite overshoot. In fact, forest need not be the offset mechanism used to illustrate the magnitude of carbon accumulation in the atmosphere [10],[34]. The area of solar panels or wind farms could serve an equivalent function in the calculation of the carbon footprint, showing the degree to which these offset mechanisms fall short of bringing net additions of carbon to the atmosphere down to zero. In conclusion, the EF's carbon footprint, as currently constructed, is an unreliable and impractical illustration of human demands on the biosphere in general and carbon emissions in particular. Hence, conclusions using the EF to assert how many planets we are using or to comment on the sustainability of human populations—current or projected—are misplaced [35],[36]. Clearly, anthropogenic emissions of greenhouse gases are a serious problem, but these are better estimated directly [37] than by calculating a “number of planets” needed to offset emissions.

## Policy Utility of the Ecological Footprint

The global ecological overshoot shown in EF calculations [1] has generated an obvious question for policy-makers, scientists, and the public alike: in which ways can we change our natural resource use and land management in order to reduce, and ultimately eliminate, the global overshoot and thereby achieve sustainability? As described in this Perspective, changes to the management or distribution of croplands, grazing lands, or built-up land would have virtually no effect on global ecological overshoot or surplus. Thus, the simplest way to reduce the global ecological overshoot, “by-the-numbers,” would be to devote large tracts of land to *Eucalyptus* plantations, which have sequestration rates around 5–10 t C ha^−1^ year^−1^ in much of the tropics and subtropics, and can reach rates of up to 12 t C ha^−1^ year^−1^ in some areas [38]. In the EF accounts, this afforestation would be recorded as forest area with exceptionally high biocapacity, thereby offsetting the deficits in the carbon footprint or any other land-use type. Based on this logical interpretation of the EF methodology, less than half the area of the United States planted with eucalypts could essentially give us an EF equal to one Earth—an approach that no ecologist would recommend. This thought experiment illustrates that the EF not only fails to provide a robust measure of ecological sustainability, but also offers poor guidance for policy-makers in identifying and evaluating options to improve use and management of natural capital.

## Guidelines for Robust Ecological Indicators

The development and selection of indicators for use in environmental policy-making should be based on sound criteria, including scientific validity and policy utility [12],[39]–[41]. To elaborate on these two broad criteria, we propose a set of principles for ecological indicators informed by our analysis of the EF.

- **Indicators should illuminate pathways towards attaining sustainability goals that make both ecological and common sense.** In keeping with this principle is the premise that covering the world with eucalypt plantations is not the optimal path to sustainability. Decision makers attempting to apply the EF to guide policies and measures that will reduce the global ecological overshoot would risk perverse consequences that are antithetical to most conceptions of sustainability.
- **Indicators of the sustainability of natural capital consumption should be able to record depletion or surpluses.** In other words, assessments should consider whether stocks of natural capital are decreasing or increasing as a result of human use. The EF is unable to reflect the sustainability of croplands, built-up land, and grazing land, since these are by definition always in near balance—the footprint of consumption roughly equating biocapacity—in the EF accounts.
- **A set of indicators, each pertaining to an identifiable and quantifiable form of natural capital or ecosystem service, is likely to be more comprehensible and useful than a single aggregate index.** Logically combined sets of indicators are more likely to offer an acceptable balance between reductionism and simplicity, on the one hand, and sound theoretical and empirical grounding, on the other [12],[40]. They also allow for trade-offs between different ecosystem services or natural resources when necessary [42],[43]. The EF attempts to provide a single index by measuring a subset of net primary productivity, regardless of its source, quality, or ecological relevance. The implicit assumption is that primary productivity—biomass generated—is the key scarce resource [12],[18]; however, this aggregation is problematic. First, it implies full substitutability between primary productivity in croplands, forests, fishing grounds, and grazing lands rather than indicating whether we have sufficient supplies of food, wood products, fish, and meat, corresponding to these four land-use types in the EF accounts. More importantly from a conservation-oriented perspective, it fails to indicate whether forest area is increasing or decreasing, whether biodiversity is being lost or gained, and whether ecosystem services are improved or damaged. These global sustainability concerns are urgent and merit rigorous measurement, but their qualitative differences argue against excessive aggregation and, instead, suggest the use of more targeted metrics [44].
- **Indicators must take into account the geographical scale of the phenomena they are measuring** [45]. The EF is inconsistent across scales; its meaning on a global level—nominally whether we are or are not depleting natural capital—differs from its meaning on subglobal scales, such as in countries, where it indicates self-sufficiency and patterns of trade (e.g., balance of imports and exports).
- **Ecological indicators should, where possible, include estimates of uncertainty.** Humanity's total footprint, as calculated in the EF, is critically dependent on a single, empirically derived variable—the carbon sequestration rate—the estimation of which is highly uncertain (see Table 2). Using a single figure without an associated confidence interval gives a false impression of precision and is therefore misleading.

## Back to the Drawing Board

Simple and practical indicators of how well humanity is managing Earth's biological resources and ecosystem services are essential to improving stewardship of the Earth system in that they bridge the domains of science and policy and thereby facilitate discussion and decision-making [46]. Beyond their potential use as direct input to policy formulation, indicators also inform broader understanding of ecological risks and opportunities [39]. As such, the scientifically robust construction and presentation of ecological indicators is a matter of great importance. By understanding the strengths and weaknesses of the EF, it will be possible to better develop and select ecological indicators as ecologists and environmental scientists go back to the drawing board.
